# Reduced circulating adiponectin levels are associated with the metabolic syndrome independently of obesity, lipid indices and serum insulin levels: a cross-sectional study

**DOI:** 10.1186/s12944-016-0311-7

**Published:** 2016-08-27

**Authors:** Agathi Ntzouvani, Elisabeth Fragopoulou, Demosthenes Panagiotakos, Christos Pitsavos, Smaragdi Antonopoulou

**Affiliations:** 1 Laboratory of Biology, Biochemistry, Physiology and Microbiology, Department of Nutrition and Dietetics, School of Health Science and Education, Harokopio University, Eleftheriou Venizelou 70, Athens, 17671 Greece; 2 Department of Nutrition and Dietetics, School of Health Science and Education, Harokopio University, Athens, Greece; 3 First Cardiology Clinic, School of Medicine, National and Kapodistrian University of Athens, Athens, Greece

**Keywords:** Metabolic syndrome, Adiponectin, Interleukin-6, Mediation effect

## Abstract

**Background:**

Given the increasing rate of overweight and the burden of metabolic syndrome (MetS) on cardiovascular disease development, better understanding of the syndrome is of great importance. Therefore, the objectives were to examine whether interleukin-6 (IL-6) and adiponectin are associated with MetS, and whether this association is mediated by components of the MetS.

**Methods:**

During 2011–2012, 284 individuals (159 men, 53 ± 9 years, 125 women 52 ± 9 years) without cardiovascular disease, type 1 diabetes mellitus, high-grade inflammatory disease, living in the greater Athens area, Greece, participated in clinical examination. Adiponectin and IL-6 were measured in fasting plasma samples. MetS was defined according to the International Diabetes Federation (IDF) and the American Heart Association/National Heart, Lung, and Blood Institute (AHA/NHLBI) criteria.

**Results:**

MetS was present in 37 % (IDF) and 33 % (AHA/NHLBI) of the study population (*P* < 0.001). Adiponectin was inversely associated with MetS (odds ratio, 95 % confidence interval: 0.829, 0.762- 0.902 for MetS-IDF, and 0.840, 0.772- 0.914 for MetS-AHA/NHLBI). Body mass index (BMI), waist circumference, high density lipoprotein (HDL)-cholesterol, triglyceride and insulin concentration mediated the association between adiponectin and MetS-IDF (z-test, standard error, *P*-value: 2.898, 0.012, 0.004, for BMI; 2.732, 0.012, 0.006 for waist circumference; 2.388, 0.011, 0.017 for HDL-cholesterol; 2.163, 0.010, 0.031 for triglyceride; 2.539,  0.010,  0.011 for insulin). Similarly, BMI, waist circumference, HDL-cholesterol and insulin concentration mediated the association between adiponectin and MetS-AHA/NHLBI (z-test, standard error, *P*-value: 2.633, 0.011, 0.008 for BMI; 2.441, 0.011, 0.015 for waist circumference; 1.980, 0.010, 0.048 for HDL-cholesterol; 2.225, 0.009, 0.026 for insulin). However, adiponectin remained significantly associated with MetS. IL-6 was not significantly associated with MetS.

**Conclusion:**

MetS components, in particular obesity and lipid indices, as well as serum insulin levels, mediate the association between adiponectin and MetS as defined by both the IDF and AHA/NHLBI criteria.

## Background

Metabolic syndrome (MetS) is a cluster of factors of clinical importance that increases the risk of cardiovascular disease (CVD). These factors are widely accepted indices of obesity, metabolic function and blood pressure homeostasis [1]. Cardiovascular disease (CVD) incidence has been recently associated with dyslipidemia, diabetes mellitus and hypertension in a cohort study of CVD epidemiology in Greece [2]. Several organizations formulated simple criteria for the diagnosis of MetS in the clinical practice in order to modify risk factors for CVD development through lifestyle changes. The World Health Organization, WHO, (1998) consultation group, the European Group for Study of Insulin Resistance, EGIR, (1999), and the American Association of Clinical Endocrinologists, AACE, (2003) emphasized insulin resistance as the underlying cause of MetS and required evidence of insulin resistance for diagnosis. The National Cholesterol Education Program Adult Treatment Panel III, NCEP ATP III, (2001) aimed at identifying people at higher long-term risk for atherosclerotic cardiovascular disease who deserved lifestyle intervention to reduce risk. The International Diabetes Federation, IDF, writing group (2005) considered that abdominal obesity is strongly correlated with insulin resistance, and made the presence of abdominal obesity a prerequisite for the diagnosis of MetS. The American Heart Association/National Heart, Lung, and Blood Institute, AHA/NHLBI, (2005) scientific statement revised the NCEP ATP III criteria as regards the threshold for impaired fasting glucose. Neither the NCEP ATP III nor the AHA/NHLBI criteria drew conclusions on mechanistic pathogenesis [3].

The occurrence of MetS has been characterized as a global epidemic. The two most widely used definitions of MetS are based on the NCEP ATP III (2001) and the IDF (2005) criteria [1]. The prevalence of MetS in the Greek population was evaluated in two population-based epidemiological studies, the ATTICA study [4] and the MetS Greece Study [5]. The prevalence of MetS was 23.6 % [5], according to the NCEP ATP III definition, and 48.9 % according to the IDF definition [6]. The most prevalent abnormality among subjects with the MetS was obesity, particularly abdominal obesity. Abdominal obesity is considered the predominant underlying cause of MetS and is associated with both insulin resistance and low-grade chronic inflammation [3]. Waist circumference is a widely used index of abdominal obesity, and was found to be a better predictor of MetS compared with BMI, waist-to-hip ratio and waist-to-height ratio [7].

Metabolic health has been associated with lower concentrations of pro-inflammatory cytokines (e.g. IL-6), and higher concentrations of anti-inflammatory adipokines (e.g. adiponectin) in both obese and non-obese adults [8]. Presence of MetS and its components have been associated with increased levels of IL-6 and decreased levels of adiponectin [3, 9]. A cross-sectional data analysis from the Diet and Omega-3 Intervention Trial on Atherosclerosis (DOIT) showed that serum levels of IL-6 were significantly higher in subjects with MetS compared to those without MetS, but there was no significant association between IL-6 and increasing MetS components [10]. On the contrary, the proportion of subjects with MetS, declined across sex-specific adiponectin quartiles in the context of the Carotid Ultrasound Disease Assessment Study [11].

Given the increasing rates of overweight and obesity, as well as the burden of MetS on cardiovascular disease development, better understanding of the syndrome is of great importance. Thus, the present study evaluated the prevalence of MetS in a sample of the Greek population, using two definitions which include an index of abdominal obesity and differ only by the waist circumference criteria. The hypothesis was that adiponectin and IL-6 plasma concentration is associated with MetS through its components. Therefore, the aims of the present study were to examine i) whether IL-6 or adiponectin concentration is associated with MetS, and ii) whether this association is mediated by components of the MetS. The ability of IL-6 or adiponectin concentration in identifying individuals with MetS was also evaluated.

## Methods

### Participants

**Fig. 1:**
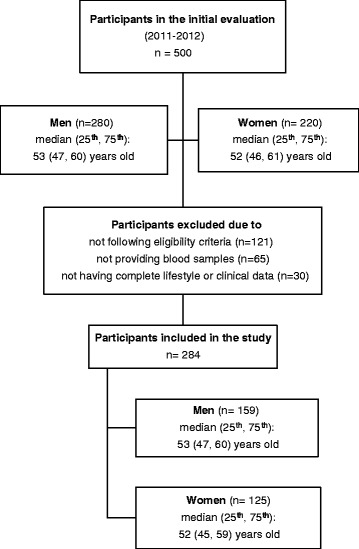
Study flowchart

This was a cross-sectional study carried out in the greater area of Athens (78 % urban and 22 % rural regions) during 2011–2012. The study population consisted of individuals aged > 30 years from the general population. Participants responded to an invitation to health evaluation which was published at the participants’ workplace. Five hundred individuals participated in the initial evaluation (Fig. 1). The sampling was based on a feasibility basis, and the evaluation was performed at each participant’s workplace or home by trained personnel (cardiologists, general practitioners, dietitians and nurses).

Participants diagnosed with cardiovascular disease (i.e. myocardial infarction, angina pectoris, other identified forms of ischemia; coronary revascularization: coronary artery bypass surgery and percutaneous coronary intervention, heart failure of different types, chronic arrhythmias, or stroke) were excluded from the study. Other exclusion criteria were presence of high-grade chronic inflammatory disease (e.g. rheumatoid arthritis, inflammatory bowel disease, atopic dermatitis, and asthma), viral infections, cold or flu, acute respiratory infection, dental problems, any type of surgery the month preceding the study, and type 1 diabetes mellitus.

Two hundred eighty four participants who were eligible to participate in the study and had complete lifestyle, clinical and biochemical data were included in the present study (Fig. 1); 159 participants were men (53 ± 9 years) and 125 were women (52 ± 9 years). No significant differences were observed between participants who were finally included in the study and the rest of the participants who were excluded, as regards age and sex (*P* > 0.30, for all).

### Lifestyle evaluation

Dietary habits were evaluated with a validated semi-quantitative food-frequency questionnaire [12]; overall dietary habits were evaluated using the MedDietScore (range 0–55) that incorporates the inherent characteristics of the Mediterranean diet [13]. Smokers were defined as those who were smoking at least one cigarette per day during the past year or had recently stopped smoking (within the last 12 months); the rest of the participants were defined as non-smokers. Passive smokers were defined as those who were exposed to other peoples’ smoke for more than 30 min/day and more than 5 days/week. The criteria for defining passive smoking were based on literature addressing the biologic effects of second-hand smoke on the cardiovascular system [14, 15]. Physical activity level was evaluated with the International Physical Activity Questionnaire (IPAQ), modified and adapted for the Greek population. Physical activity was classified as vigorous, moderate and walking physical activity and expressed in MET-minutes per week (MET.min.wk^−1^). Total physical activity level and sitting hours per day were also evaluated [16].

### Clinical evaluation

Resting blood pressure was measured twice on the right arm with an electronic monitor device. All participants were at least 30 min at rest before measurement which was performed in sitting position. Diagnosis and current medication treatment for hypertension, hyperlipidemia and type 2 diabetes mellitus were recorded in a self-administered questionnaire. Waist circumference (in centimeters, cm) was measured in the middle between the 12^th^ rib and the iliac crest. Hip circumference (in cm) was measured around the buttocks at the level of the maximum extension. Height was measured to the nearest 0.5 cm, without shoes, back against the wall tape, and eyes looking straight ahead. Weight was measured with a lever balance, to the nearest 100 g, without shoes, in light undergarments. BMI was calculated as weight (in kilograms, kg) divided by height (in meters squared, m^2^). Overweight was defined as BMI between 25 and 29.9 kg/m^2^, while obesity as BMI greater than 29.9 kg/m^2^, based on the WHO criteria.

### Blood collection and biochemical analyses

Venous blood samples were collected between 08:00 and 10:00, after 12 h overnight fast, with the participant in sitting position. Instructions about the preparation before blood collection were given to each participant either by telephone or by e-mail. Fasting serum was obtained by collecting blood into silicone coated Vacutainer Tubes (Becton Dickinson) with clot activator. Blood was allowed to clot at room temperature (18 – 25 °C) for 60 min and immediately centrifuged for 10 min at 1,500xg before isolation of the serum fraction. Fasting plasma was obtained by collecting blood into K_2_-EDTA (EDTA-dipotassium salt) Vacutainer Tubes (Becton Dickinson); the final EDTA concentration in the samples was 4 mmol/L. The EDTA blood samples were centrifuged within 60 min at room temperature for 10 min at 1,500xg. Plasma and serum aliquots were stored at −80 °C until use.

Serum total cholesterol, HDL-cholesterol, triglyceride, and glucose concentration were measured on a COBAS 8000/ ROCHE analyzer, based on colorimetric detection. The CHOD-PAP method was applied for total cholesterol (2.06 % intra-assay coefficient of variation-CV, 0.94 % inter-assay CV) and HDL cholesterol (1.50 % intra-CV, 0.80 % inter-CV), the GPO-PAP method for triglycerides (1.80 % intra-CV, 1.98 % inter-CV), and the GOD-PAP method for glucose (1.97 % intra-CV, 1.28 % inter-CV). All measurements were carried out at the same laboratory (BIOMED S.A., accreditation standard ELOT EN ISO 15189, Hellenic Accreditation System – E.SY.D.). None of the study participants had triglyceride values >4.5 mmol/L. LDL-cholesterol was estimated with the Friedewald equation: (*total cholesterol*) – (*HDL cholesterol*) – (*triglycerides*/2.2) [17]. All biochemical indices were measured in duplicate and are expressed in mmol/L.

Serum insulin concentration was measured on a TOSOH AIA-600 II automated enzyme immunoassay analyzer using a two-site immune-enzymometric assay. The intra- and inter-assay CVs were <3 %. Insulin concentration is expressed in mU/L. IL-6 and adiponectin concentrations were measured in plasma EDTA samples. IL-6 was measured by a commercially available ELISA method (Quantikine HS, R&D Systems Europe Ltd., Abingdon, U.K.) with an assay range of 0.156 -10 pg/ml. Adiponectin was measured by a commercially available ELISA method (Quantikine, R&D Systems Europe Ltd., Abingdon, U.K.) with an assay range of 3.9 - 250 ng/ml. The intra- and inter-assay CVs were <10 % for IL-6 and <7 % for adiponectin. Il-6 and adiponectin concentrations are expressed in pg/ml and μg/ml, respectively.

### Definition of metabolic syndrome

Metabolic syndrome was defined by the AHA/NHLBI and the IDF criteria [3]. According to the AHA/NHLBI definition, the diagnosis of MetS is established when 3 of 5 factors are present: abdominal obesity, elevated triglycerides, reduced HDL cholesterol, elevated blood pressure, elevated fasting glucose or type 2 diabetes mellitus. The diagnostic criteria are: waist circumference ≥102 cm for men and ≥88 cm for women, triglycerides ≥1.7 mmol/L, HDL-cholesterol <1.03 mmol/L for men and <1.3 mmol/L for women, systolic blood pressure (SBP) ≥130 mmHg or diastolic blood pressure (DBP) ≥85 mmHg, and fasting blood glucose ≥5.6 mmol/L. Participants on drug treatment for elevated triglycerides, reduced HDL-cholesterol, elevated blood pressure or hyperglycemia were considered as meeting the aforementioned criteria, respectively. Presence of MetS was also defined by the IDF criteria. According to the IDF, one must have abdominal obesity (waist circumference ≥94 cm for men, and ≥80 cm for women of Europid origin) or BMI ≥30 kg/m^2^ and any two of the other risk factors mentioned in the AHA/NHLBI definition.

### Bioethics

The study adhered to the Declaration of Helsinki principles, and it was approved by the Bioethics Committee of Harokopio University, Athens. Participants were informed about the aims and the procedure of the study and they provided their written consent prior to the collection of any information.

### Statistical analysis

The prevalence of MetS was determined as frequencies, using both definitions. Continuous variables are expressed as median and interquartile range (IQR). Categorical variables are expressed as frequencies. Normal distribution of continuous variables was tested with the Kolmogorov-Smirnov test and P-P plots. Adiponectin and IL-6 had a rightly skewed distribution and the values were log-transformed (log10). The association of continuous variables (i.e. age, HDL-cholesterol, LDL-cholesterol, triglycerides, glucose, insulin, SBP, DBP, BMI, waist circumference, IL-6 and adiponectin) with MetS was evaluated using the non-parametric Kolmogorov - Smirnov Z-test (due to the skewed distribution of the variables), whereas the Pearson Chi-Square test was used for the categorical variables (i.e. sex and medical treatment for hypertension, hypercholesterolemia, type 2 diabetes mellitus). Pearson’s unadjusted and partial (adjusted for sex and age) correlation coefficient (r) was applied to evaluate correlations between adiponectin, IL-6 and MetS components (i.e. waist circumference, BMI, HDL-cholesterol, triglycerides, glucose, insulin, SBP and DBP). Linear regression was applied, where adiponectin or IL-6 was the dependent variable and sex, age and MetS components were the independent variables; results are presented as beta coefficients and SE. The collinearity statistics (tolerance and variance inflation factor, VIF) showed that there was no problem with multicollinearity. Logistic regression was applied, where MetS was the dependent variable and adiponectin or IL-6 was the independent variable, and adjusted for sex and age, and further adjusted for BMI, waist circumference, HDL-cholesterol, triglyceride and insulin concentration in order to test for the potential mediating effect of these factors as regards the adiponectin - MetS and IL-6-MetS association. Results are expressed as odds ratio (OR) and the corresponding 95 % confidence interval (CI). The Sobel Test was used to determine whether the indirect association between the independent variable (i.e., IL-6 or adiponectin) and the dependent variable (i.e., MetS) via the mediator is significantly different from zero [18]. The test was performed online http://quantpsy.org/sobel/sobel.htm. The receiver operating characteristic (ROC) analysis was used to assess the ability of adiponectin to discriminate between participants with MetS and participants without MetS after adjustment for sex, age, and further adjustment for BMI, waist circumference and insulin concentration. Results are expressed as the area under the ROC curve (AUC) and the corresponding 95 % CI. The AUC provides a scale from 0.5 to 1.0 (i.e. 0.5 represents random chance and 1.0 indicates perfect discrimination) by which to appraise the accuracy of adiponectin. ROC analysis was performed using R (version 3.2.4 for Windows), package “pROC” [19]. SPSS version 21 (Statistical Package for Social Sciences, SPSS Inc., Chicago, IL, USA) was used for all other statistical analyses. All reported *P*-values are based on two-sided tests, and statistical significance was set at *P* < 0.05.

## Results

### Prevalence of MetS and comparison for lifestyle, clinical and biochemical factors

Table 1 shows the general characteristics of the study population, and the comparison between participants with MetS and participants without MetS. The prevalence of MetS was 37 % of the total study population (32 women and 74 men) according to the IDF criteria, and 33 % (29 women and 66 men) according to the AHA/NHLBI criteria. The prevalence of MetS differed significantly between the two definitions (*P* < 0.001). Participants with MetS did not differ significantly as regards current smoking habits (smokers vs. non-smokers, cigarettes per day and years of smoking), passive smoking (passive smokers vs. non-passive smokers), physical activity level (MET.min.wk^−1^), sitting hours (h/day) or dietary habits (portions of food group consumption per wk and MedDietScore) compared with participants without MetS as defined by either the IDF or the AHA/NHLBI criteria (data not shown).

**Table 1:** Clinical and biochemical factors in the total study sample, participants without metabolic syndrome and participants with metabolic syndrome

		IDF (n, %)		AHA/NHLBI (n, %)	
	All	no MetS (178, 63)	MetS (106, 37)	no MetS (189, 67)	MetS (95, 33)
Age (years)	52.00 (46.00, 59.88)	51.00 (45.00, 56.00)	56.50 (49.00, 62.25)^b^	51.00 (45.00, 56.50)	56.00 (49.00, 62.00)^b^
Sex (men, women), n (%)	159 (56)/ 125(44)	85 (48)/ 93 (52)	74 (70)/ 32 (30)^a^	93 (49)/ 96 (51)	66 (69)/ 29 (31)^a^
HDL-cholesterol (mmol/L)	1.29 (1.08, 1.51)	1.38 (1.21, 1.58)	1.09 (0.94, 1.29)^b^	1.38 (1.20, 1.58)	1.08 (0.94, 1.25)^b^
LDL-cholesterol (mmol/L)	3.43 (2.91, 4.04)	3.46 (2.97, 4.06)	3.30 (2.73, 3.92)	3.45 (2.94, 3.95)	3.38 (2.90, 4.10)
Triglycerides (mmol/L)	1.15 (0.86, 1.62)	1.05 (0.76, 1.30)	1.61 (1.14, 2.18)^b^	1.05 (0.77, 1.30)	1.70 (1.13, 2.33)^b^
Glucose (mmol/L)	4.99 (4.72, 5.35)	4.90 (4.69, 5.17)	5.22 (4.86, 5.71)^b^	4.90 (4.68, 5.17)	5.24 (4.90, 5.82)^b^
Insulin (mU/L)	8.20 (5.80, 11.85)	6.80 (4.90, 9.20)	11.30 (8.65, 14.00)^b^	6.80 (5.00, 9.05)	11.80 (9.10, 14.87)^b^
Medical treatment for:					
Hypertension (no/yes), n (%)	213 (75)/ 49 (17.3)	152 (85.4)/ 14 (7.9)	61 (57.5)/ 35 (33)^a^	155 (82)/ 20 (10.6)	58 (61.1)/ 29 (30.5)^a^
Hypercholesterolemia (no/yes), n (%)	161 (56.7)/ 109 (38.4)	118 (66.3)/ 52 (29.2)	43 (40.6)/ 57 (53.8)^a^	122 (64.6)/ 57 (30.2)	39 (41.1)/ 52 (54.7)^a^
Type 2 diabetes mellitus (no/yes), n (%)	250 (88)/ 14 (4.9)	165 (92.7)/ 2 (1.1)	85 (80.2)/ 12 (11.3)^a^	175 (92.6)/ 2 (1.1)	75 (78.9)/ 12 (12.6)^a^
SBP (mmHg)	123.00 (114.00, 133.50)	118.00 (109.75, 127.00)	133.00 (124.75, 138.00)^b^	118.50 (110.00, 128.00)	133.00 (125.25, 138.00)^b^
DBP (mmHg)	79.00 (70.00, 86.00)	75.25 (69.00, 82.40)	84.00 (77.00, 90.00)^b^	76.00 (69.00, 83.00)	85.00 (77.00, 90.00)^b^
BMI (kg/m^2^)	27.25 (24.73, 31.00)	25.40 (23.90, 29.00)	29.80 (27.20, 32.40)^b^	25.70 (23.95, 28.85)	30.00 (28.00, 32.70)^b^
Waist circumference (cm)	96.00 (86.00, 106.00)	90.00 (81.25, 98.88)	103.00 (97.75, 111.25)^b^	90.00 (82.00, 99.00)	105.00 (98.00, 112.00)^b^
IL-6 plasma concentration (pg/mL)	1.621 (0.983, 2.696)	1.392 (0.897, 2.209)	2.152 (1.274, 3.026)^b^	1.394 (0.897, 2.329)	2.099 (1.391, 3.036)^b^
Adiponectin plasma concentration (μg/mL)	7.419 (4.763, 10.516)	8.471 (5.883, 12.944)	5.546 (3.799, 8.693)^b^	8.473 (5.680, 12.579)	5.476 (4.072, 8.454)^b^

Quantitative variables are expressed as median (25th, 75th IQR)

^a^Pearson Chi-Square, *P* ≤ 0.001 for comparison between no MetS and MetS separately for two definitions of MetS

^b^Kolmogorov-Smirnov Z Test, *P* ≤ 0.001 for comparison between no MetS and MetS separately for two definitions of MetS

According to the unadjusted bivariate associations, adiponectin was positively correlated with HDL-cholesterol (*r* = 0.385, *P* < 0.001) and negatively correlated with triglycerides (*r* = −0.362, *P* < 0.001), glucose (*r* = −0.219, *P* < 0.001), insulin (*r* = −0.312, *P* < 0.001), SBP (*r* = −0.123, *P* < 0.05), DBP (*r* = −0.128, *P* < 0.05), BMI (*r* = −0.171, *P* < 0.01) and waist circumference (*r* = −0.391, *P* < 0.001). On the contrary, IL-6 was negatively correlated with HDL-cholesterol (*r* = −0.308, *P* < 0.001) and positively correlated with triglycerides (*r* = 0.219, *P* < 0.001), insulin (*r* = 0.125, *P* < 0.05), SBP (*r* = 0.124, *P* < 0.05), BMI (*r* = 0.267, *P* < 0.001) and waist circumference (*r* = 0.298, *P* < 0.001). Age was significantly correlated with triglyceride, glucose and insulin concentration (*r* = 0.164, *P* < 0.01, *r* = 0.265, *P* < 0.001, and *r* = 0.132, *P* < 0.05 respectively) in the total study population. Age was also significantly correlated with SBP and waist circumference (*r* = 0.346, *P* < 0.001 and *r* = 0.132, *P* <0.05, respectively). Men had significantly higher triglyceride, glucose and insulin concentration, SBP and DBP, BMI and waist circumference than women. On the contrary, men had lower HDL-cholesterol than women (*P* < 0.001, for all). After adjustment for age and sex, the association between adiponectin and HDL-cholesterol (*r* = 0.282), triglycerides (*r* = −0.319), glucose (*r* = −0.140), insulin (*r* = −0.305), BMI (*r* = −0.161) and waist circumference (*r* = −0.208) remained statistically significant. The association between IL-6 and HDL-cholesterol (*r* = −0.335), triglycerides (*r* = 0.199), insulin (*r* = 0.130), BMI (*r* = 0.264) and waist circumference (*r* = 0.333) remained statistically significant after adjustment for sex and age. There was no significant association between adiponectin and IL-6 either in the unadjusted or the adjusted model.

In the multivariate adjusted models, adiponectin was significantly associated with serum triglyceride concentration (Table 2; Model 1 and Model 2) and waist circumference (Table 2; Model 2). Insulin was an independent associate of adiponectin (Table 2; Model 3, β = −0.164, *P* = 0.006, and Model 4, β = −0.148, *P* = 0.011) after adjusting for sex, age and MetS components. Waist circumference did not remain an independent associate of adiponectin after adjustment for insulin (Table 2; Model 4). IL-6 was significantly associated with HDL-cholesterol, BMI and waist circumference in the multivariate adjusted models (Table 3). Insulin was not an independent associate of IL-6 after adjustment for sex, age and MetS components (Table 3; Model 3 and Model 4).

**Table 2:** Multivariate adjusted associations between metabolic syndrome components and log_10_(adiponectin)

	Model 1	*R* ^2^ = 0.376				Model 3	*R* ^2^ = 0.393			
	B	SE	β	t	*P*	B	SE	β	t	*P*
Sex (women/men)	−0.194	0.025	−0.405	−7.618	<0.001	−0.189	0.025	−0.395	−7.456	<0.001
Age (years)	0.005	0.001	0.210	4.061	<0.001	0.005	0.001	0.202	3.942	<0.001
HDL-cholesterol (mmol/L)	0.002	0.001	0.119	1.931	0.055	0.002	0.001	0.119	1.948	0.053
Triglycerides (mmol/L)	−0.001	0.000	−0.213	−3.538	<0.001	−0.001	0.000	−0.169	−2.736	0.007
Glucose (mmol/L)	−0.001	0.001	−0.046	−0.842	0.401	−0.001	0.001	−0.040	−0.741	0.459
Body mass index (kg/m^2^)	−0.004	0.003	−0.074	−1.432	0.153	0.000	0.003	−0.004	−0.074	0.941
Insulin (mU/L)	-	-	-	-	-	−0.007	0.002	−0.164	−2.755	0.006
	Model 2	*R* ^2^ = 0.380				Model 4	*R* ^2^ = 0.394			
	B	SE	β	t	*P*	B	SE	β	t	*P*
Sex (women/men)	−0.172	0.028	−0.359	−6.206	<0.001	−0.179	0.028	−0.375	−6.410	<0.001
Age (years)	0.006	0.001	0.211	4.103	<0.001	0.005	0.001	0.205	3.999	<0.001
HDL-cholesterol (mmol/L)	0.002	0.001	0.119	1.954	0.052	0.002	0.001	0.117	1.912	0.057
Triglycerides (mmol/L)	−0.001	0.000	−0.201	−3.313	0.001	−0.001	0.000	−0.165	−2.670	0.008
Glucose (mmol/L)	−0.001	0.001	−0.043	−0.784	0.434	−0.001	0.001	−0.037	−0.674	0.501
Waist circumference (cm)	−0.002	0.001	−0.117	−1.975	0.049	−0.001	0.001	−0.054	−0.848	0.398
Insulin (mU/L)	-	-	-	-	-	−0.006	0.002	−0.148	−2.558	0.011

**Table 3:** Multivariate adjusted associations between metabolic syndrome components and log_10_(IL-6)

	Model 1	*R* ^2^ = 0.199				Model 3	*R* ^2^ = 0.220			
	B	SE	β	t	*P*	B	SE	β	t	*P*
Sex (women/men)	−0.079	0.040	−0.117	−1.951	0.052	−0.090	0.040	−0.134	−2.224	0.027
Age (years)	0.008	0.002	0.211	3.605	<0.001	0.008	0.002	0.216	3.720	<0.001
HDL-cholesterol (mmol/L)	−0.008	0.002	−0.265	−3.817	<0.001	−0.008	0.002	−0.286	−4.110	<0.001
Triglycerides (mmol/L)	0.000	0.000	0.067	0.985	0.326	0.000	0.000	0.039	0.564	0.573
Glucose (mmol/L)	−0.002	0.002	−0.050	−0.803	0.422	−0.002	0.002	−0.059	−0.953	0.342
Body mass index (kg/m^2^)	0.013	0.004	0.201	3.483	0.001	0.011	0.004	0.172	2.793	0.006
Insulin (mU/L)	-	-	-	-	-	0.005	0.004	0.084	1.296	0.196
	Model 2	*R* ^2^ = 0.236				Model 4	*R* ^2^ = 0.253			
	B	SE	β	t	*P*	B	SE	β	t	*P*
Sex (women/men)	−0.171	0.043	−0.256	−3.991	<0.001	−0.179	0.043	−0.268	−4.123	<0.001
Age (years)	0.007	0.002	0.205	3.586	<0.001	0.008	0.002	0.214	3.740	<0.001
HDL-cholesterol (mmol/L)	−0.007	0.002	−0.261	−3.884	<0.001	−0.008	0.002	−0.287	−4.242	<0.001
Triglycerides (mmol/L)	0.000	0.000	0.040	0.591	0.555	0.000	0.000	0.027	0.397	0.692
Glucose (mmol/L)	−0.002	0.002	−0.067	−1.110	0.268	−0.002	0.002	−0.072	−1.184	0.238
Waist circumference (cm)	0.008	0.002	0.320	4.872	<0.001	0.008	0.002	0.312	4.396	<0.001
Insulin (mU/L)	-	-	-	-	-	0.001	0.004	0.025	0.382	0.703

### Assessing the potential mediation effect of insulin, BMI, waist circumference, HDL-cholesterol and triglycerides

Plasma adiponectin concentration was significantly associated with MetS (OR, 95 % CI: 0.829, 0.762- 0.902 for MetS-IDF, and 0.840, 0.772- 0.914 for MetS-AHA/NHLBI) (Table 4), whereas plasma IL-6 concentration was not significantly associated with MetS (Table 5). Adiponectin remained significantly associated with MetS after controlling for insulin, BMI, waist circumference, HDL-cholesterol or triglyceride concentration. However, adiponectin was not significantly associated with MetS-AHA/NHLBI after controlling for triglyceride concentration (Table 4). Results of the Sobel Test showed that the indirect association between adiponectin and MetS-IDF was significantly different from zero after controlling for insulin (z-test = 2.539, SE = 0.010, *P* = 0.011), BMI (z-test = 2.898, SE = 0.012, *P* = 0.004), waist circumference (z-test = 2.732, SE = 0.012, *P* = 0.006), HDL-cholesterol (z-test = 2.388, SE = 0.011, *P* = 0.017) or triglyceride concentration (z-test = 2.163, SE = 0.010, *P* = 0.031). Similarly, the indirect association between adiponectin and MetS-AHA/NHLBI was significantly different from zero after controlling for insulin (z-test = 2.225, SE = 0.009, *P* = 0.026), BMI (z-test = 2.633, SE = 0.011, *P* = 0.008), waist circumference (z-test = 2.441, SE = 0.011, *P* = 0.015) or HDL-cholesterol (z-test = 1.980, SE = 0.010, *P* = 0.048).

**Table 4:** Association between plasma adiponectin concentration and metabolic syndrome, and the mediation effect of MS components

		OR (95 % CI)	OR (95 % CI)	OR (95 % CI)	OR (95 % CI)	OR (95 % CI)	OR (95 % CI)
MetS-IDF	Age (years)	1.087 (1.052- 1.123)	1.084 (1.048- 1.122)	1.088 (1.051- 1.126)	1.084 (1.045- 1.124)	1.089 (1.051- 1.128)	1.075 (1.037- 1.113)
	Sex (men)	0.757 (0.404- 1.418)	0.685 (0.353-1.330)	0.652 (0.330- 1.290)	1.656 (0.784- 3.497)	0.976 (0.487- 1.956)	0.743 (0.372- 1.486)
	Adiponectin (μg/ml)	0.829 (0.762- 0.902)	0.870 (0.797-0.949)	0.837 (0.764- 0.916)	0.846 (0.769- 0.930)	0.875 (0.798- 0.958)	0.894 (0.818- 0.977)
	Insulin (mU/L)	-	1.131 (1.069-1.197)	-	-	-	-
	BMI (kg/m^2^)	-	-	1.193 (1.113- 1.278)	-	-	-
	Waist circumference (cm)	-		-	1.098 (1.064- 1.133)	-	-
	HDL-cholesterol (mmol/L)	-		-	-	0.904 (0.873- 0.937)	-
	Triglycerides (mmol/L)	-		-	-	-	1.020 (1.013- 1.027)
MetS-AHA/NHLBI	Age (years)	1.082 (1.047- 1.117)	1.080 (1.043-1.118)	1.083 (1.046- 1.122)	1.078 (1.039- 1.117)	1.086 (1.047- 1.126)	1.070 (1.031- 1.110)
	Sex (men)	0.803 (0.425- 1.517)	0.720 (0.366-1.418)	0.667 (0.331- 1.343)	1.811 (0.844- 3.886)	1.103 (0.534- 2.276)	0.779 (0.377- 1.609)
	Adiponectin (μg/ml)	0.840 (0.772- 0.914)	0.887 (0.813-0.969)	0.850 (0.776- 0.931)	0.861 (0.783- 0.947)	0.897 (0.817- 0.985)	0.921 (0.842- 1.007)
	Insulin (mU/L)	-	1.147 (1.083-1.215)	-	-	-	-
	BMI (kg/m^2^)	-	-	1.212 (1.129- 1.302)	-	-	-
	Waist circumference (cm)	-		-	1.105 (1.070- 1.141)	-	-
	HDL-cholesterol (mmol/L)	-		-	-	0.884 (0.850- 0.919)	-
	Triglycerides (mmol/L)	-		-	-	-	1.023 (1.016- 1.031)

**Table 5:** Association between plasma IL-6 concentration and metabolic syndrome, and the mediation effect of MS components

		OR (95 % CI)	OR (95 % CI)	OR (95 % CI)	OR (95 % CI)	OR (95 % CI)	OR (95 % CI)
MetS-IDF	Age (years)	1.072 (1.040- 1.105)	1.072 (1.038-1.108)	1.076 (1.042- 1.111)	1.072 (1.037- 1.109)	1.091 (1.053- 1.129)	1.068 (1.032- 1.105)
	Sex (men)	0.357 (0.205- 0.624)	0.405 (0.223-0.738)	0.345 (0.190- 0.626)	0.957 (0.481- 1.907)	0.690 (0.364- 1.309)	0.493 (0.262- 0.927)
	IL-6 (pg/ml)	1.086 (0.975- 1.210)	1.051 (0.935-1.182)	1.054 (0.934- 1.191)	1.021 (0.894- 1.166)	0.968 (0.853- 1.099)	1.033 (0.907- 1.176)
	Insulin (mU/L)	-	1.154 (1.090-1.221)	-	-	-	-
	BMI (kg/m^2^)	-	-	1.139 (1.073- 1.209)	-	-	-
	Waist circumference (cm)	-	-	-	1.097 (1.065- 1.131)	-	-
	HDL-cholesterol (mmol/L)	-	-	-	-	0.893 (0.861- 0.925)	-
	Triglycerides (mmol/L)	-	-	-	-	-	1.023 (1.015- 1.030)
MetS-AHA/NHLBI	Age (years)	1.066 (1.034- 1.098)	1.067 (1.033-1.103)	1.072 (1.038- 1.108)	1.068 (1.031- 1.105)	1.090 (1.051- 1.131)	1.063 (1.026- 1.102)
	Sex (men)	0.395 (0.224- 0.695)	0.456 (0.246-0.844)	0.372 (0.201- 0.689)	1.171 (0.573- 2.395)	0.923 (0.467- 1.824)	0.579 (0.299- 1.123)
	IL-6 (pg/ml)	1.064 (0.954- 1.186)	1.024 (0.907-1.156)	1.019 (0.894- 1.162)	0.983 (0.854- 1.131)	0.918 (0.805- 1.048)	0.995 (0.870- 1.139)
	Insulin (mU/L)	-	1.168 (1.103-1.237)	-	-	-	-
	BMI (kg/m^2^)	-	-	1.167 (1.094- 1.244)	-	-	-
	Waist circumference (cm)	-	-	-	1.110 (1.075- 1.146)	-	-
	HDL-cholesterol (mmol/L)	-	-	-	-	0.865 (0.830- 0.902)	-
	Triglycerides (mmol/L)	-	-	-	-	-	1.026 (1.018- 1.034)

### Accuracy of factors associated with the identification of MetS

The AUC and the 95 % CI were calculated for adiponectin after adjustment for sex, age, and further adjustment for insulin, BMI or waist circumference, in order to assess the ability of adiponectin to discriminate between participants with MetS and participants without MetS. Adiponectin performed better than chance (i.e., the AUC was significantly greater than 0.5) in classifying correctly subjects with MetS, both in the model that adjusted only for sex and age, as well as in the model that further adjusted for insulin, BMI or waist circumference (Table 6). The results were similar independently of the definition used. Since IL-6 concentration was not significantly associated with MetS, the AUC was not calculated for this biomarker.

**Table 6:** Discriminative accuracy of adiponectin, insulin, BMI and waist circumference in the prediction of prevalent metabolic syndrome

		AUC	95 % CI
MetS-IDF	Adiponectin	0.744	0.685–0.801
	Insulin	0.783	0.730–0.837
	BMI	0.752	0.688–0.815
	Waist circumference	0.798	0.743–0.853
	Adiponectin and insulin	0.813	0.760–0.858
	Adiponectin and BMI	0.793	0.734–0.850
	Adiponectin and waist circumference	0.825	0.767–0.883
	Adiponectin and insulin and BMI	0.829	0.780–0.874
	Adiponectin and insulin and waist circumference	0.858	0.814–0.896
MetS-AHA/NHLBI	Adiponectin	0.731	0.672–0.791
	Insulin	0.789	0.734–0.843
	BMI	0.758	0.696–0.817
	Waist circumference	0.803	0.746–0.859
	Adiponectin and insulin	0.818	0.763–0.862
	Adiponectin and BMI	0.791	0.732–0.849
	Adiponectin and waist circumference	0.822	0.767–0.879
	Adiponectin and insulin and BMI	0.840	0.793–0.884
	Adiponectin and insulin and waist circumference	0.860	0.813–0.899

All models were adjusted for sex and age

## Discussion

The major findings of the present study are summarized as follows. Adiponectin was significantly associated with prevalent MetS, whereas IL-6 was not significantly associated with MetS. MetS components mediated the association between adiponectin and MetS, but adiponectin remained significantly associated with MetS. Adiponectin had a high discriminative accuracy for MetS independently of the definition used. All the results were similar regardless of the definition used. Even though abdominal obesity is a prerequisite for the IDF definition and the cut-off value of waist circumference is different between the two definitions used in the present study, the results indicated that the IDF and AHA/NHLBI definitions for evaluating presence of MetS are practically identical. However, we could not draw definite conclusions based on the results of the present study due to two main limitations, i.e. the cross-sectional design of the study and the relatively small sample size. Nevertheless, the importance of the present study lies in exploring MetS beyond its clinical and biochemical constituents.

Obesity, particularly abdominal obesity, has been identified as a significant constituent of MetS. According to the IDF definition, abdominal obesity, as assessed by waist circumference, is an essential diagnostic criterion because of the strength of the evidence linking waist circumference with metabolic abnormalities [20]. Dyslipidaemia was the major metabolic abnormality and the second most frequent MetS component after abdominal obesity, found in our sample. Dyslipidaemia, which is primarily characterized by elevated plasma FFA and triglycerides, decreased levels of HDL-cholesterol, and abnormal LDL composition, is a main risk factor for CVD incidence and mortality. Normalization of fasting blood lipid levels can improve the cardiovascular risk profile of individuals. The Mediterranean dietary pattern is a lifestyle factor which consists of food groups that are already known to ameliorate dyslipidaemia and decrease the incidence of cardiovascular events, probably by means of their nutrient content, such as resveratrol and polyphenols in red wine, fish oil and proteins, polyphenols, and phytosterols in fruits and vegetables [21]. In the present study, participants with MetS did not differ significantly as regards food group consumption or their overall score of adherence to the Mediterranean dietary pattern compared with participants without MetS. However, this was a cross-sectional study, and the participants’ dietary habits were evaluated once using a semi-quantitative food-frequency questionnaire, therefore evaluation of true intake may be inaccurate due to daily and seasonal effects, as well as other personal characteristics [12].

Metabolic health has been associated with an altered secretion pattern of adipokines. Specifically, production of pro-inflammatory cytokines is enhanced, whereas production of adiponectin is inhibited in the adipose tissue in the presence of obesity [22]. Furthermore, certain adipokines, such as leptin, are mainly associated with total obesity, whereas others, such as IL-6 and adiponectin may be more closely linked with abdominal obesity [23]. It is estimated that 15–35 % of total IL-6 concentration in the circulation originates from the adipose tissue where it is produced by non-adipocytes, such as fibroplasts, endothelial cells, and monocytes [24]. Adiponectin is abundantly expressed in the white adipose tissue by mature adipocytes [25, 26], and it circulates in different oligomeric forms, i.e. low-molecular weight (LMW) trimers, medium-molecular weight (MMW) hexamers, and high-molecular weight (HMW) oligomers [27]. HMW adiponectin has been suggested as the biologically active form of adiponectin [28], and circulating HMW adiponectin, rather than total adiponectin, has been associated with insulin sensitivity [29], as well as with anteroposterior diameter of infra-renal abdominal aorta (APAO), an ultrasound early marker of atherosclerosis [30]. Nevertheless, there has been found a strong association between total and HMW adiponectin in circulation [31, 32]. In addition, both total and HMW adiponectin blood levels have been inversely associated with biomarkers of inflammation, endothelial dysfunction, and insulin resistance [33], as well as with prevalent or incident MetS [34, 35].

There has been evidence for a reciprocal association between adiponectin, pro-inflammatory cytokines (e.g. IL-6 and TNF-a), and the acute-phase reactant CRP, each regulating the expression of the others in a feedback loop [36]. IL-6 is a central mediator of the acute-phase response because it can induce the production of CRP from the liver [37] and the content of adipose tissue in IL-6 is significantly associated with the concentration of IL-6 and CRP in the circulation [38]. Adiponectin, as an anti-inflammatory adipokine, can inhibit IL-6 production [39], suppress TNF-a levels [40], and reduce IL-6-stimulated CRP secretion [41]. On the other hand, adiponectin gene expression and secretion have been found to be downregulated by IL-6, TNF-a [28], and CRP [42]. Based on the literature discussed so far, we measured total adiponectin and IL-6 as representative inflammatory indices in order to study the association with metabolic indices and the prevalence of MetS.

In the present study, adiponectin was significantly and inversely associated with waist circumference, whereas IL-6 was significantly and positively associated with BMI and waist circumference in the multivariate linear regression models. These results are in accordance with previous studies which have shown that adiponectin is inversely associated with markers of adiposity such as fat mass, BMI, waist circumference and waist-to-hip ratio in multivariate linear regression models [43–46]. Central obesity has been recognized as significant predictor of high IL-6 levels in the circulation even after adjustment for markers of lipid and glucose metabolism, blood pressure, smoking status, sex and age [47]. Abdominal obesity, assessed as waist circumference, has been found to explain most of the variance in IL-6 levels after adjustment for age, sex, smoking habits, alcohol consumption, and physical activity level [48]. In the present study, adiponectin was significantly and negatively associated with serum triglycerides in the multivariate adjusted model, and this association was attenuated, but remained significant after adjustment for serum insulin concentration. IL-6 was significantly and negatively associated with serum HDL-cholesterol in the multivariate adjusted model, but this association was not affected after further adjustment for serum insulin concentration. Previous studies have shown that adipokines are associated with insulin signalling and actions, and can therefore affect lipid metabolism. Specifically, adiponectin can have beneficial effects on lipid metabolism because of its insulin-sensitizing effects, whereas IL-6 has been involved in the dysregulation of insulin signalling and has been associated with dyslipidaemia [49–52]. Since this was a cross-sectional study, measurement of blood lipid, insulin and adipokine levels are based on a single sample, and therefore we cannot draw conclusions regarding cause-effect.

In the present study, adiponectin levels were inversely associated with prevalent MetS even after adjustment for MetS components using either definition. Similarly, previous studies have found that the odds ratio for either prevalent or incident MetS is lower for the top quartile of adiponectin compared with the bottom quartile even after adjustments for age, sex, MetS components and other MetS-related factors [11, 53–56]. In the present study, IL-6 levels were not associated with MetS. Previous study has found that IL-6 is associated with MetS, and that the top tertile of IL-6 is associated with increased odds ratio for MetS compared with the bottom tertile after adjustment for age and sex. Nevertheless, further adjustment for BMI and insulin has attenuated that association [57]. Similarly, IL-6 levels have not been associated with MetS after adjustment for age, sex and MetS components [58]. In the present study, adjustments for MetS components (i.e. BMI, waist circumference, HDL-cholesterol, triglyceride and insulin concentration) were made in order to test the potential mediating effect of these factors as regards the adiponectin-MetS association. Furthermore, the indirect association between adiponectin and MetS via the MetS components was determined in order to provide for an explanation regarding the association observed between adiponectin and MetS. As far as we are aware of, this is the first study which examines the mediation effect of MetS components in the association between adiponectin and MetS found in previous studies and in the present study as well.

In the present study, adiponectin had a high discriminative accuracy for MetS after adjustment for sex, age, and after further adjustment for BMI, waist circumference and insulin concentration, regardless of the definition used. Previous studies have also found that adiponectin has a high discriminative accuracy for MetS. In particular, plasma adiponectin quartiles have been found to classify correctly subjects with prevalent MetS after adjustment for sex, age, BMI, and MetS-related factors [11]. Similarly, plasma adiponectin has been found to have high discriminative accuracy for prevalent MetS, and it has been observed to be similar to the discriminative accuracy of BMI in age and sex adjusted models [53]. Finally, a previous study has found that adiponectin levels have a high discriminative accuracy for incident MetS [56]. As far as we are aware of, this is the first study that used two definitions in order to diagnose presence of MetS, and that evaluated the discriminative accuracy of adiponectin in classifying correctly subjects with MetS by both definitions.

Limitations of the present study have to be taken into account. Firstly, this was a cross-sectional study which does not allow for conclusions regarding causality. Secondly, this study included middle-aged white individuals, without prevalent cardiovascular disease. Caution is thus needed in the extrapolation of the findings to other populations, i.e. younger, of other ethnicity or with cardiovascular disease. Moreover, type 2 diabetes mellitus and impaired fasting glucose were less prevalent than dyslipidemia or hypertension in the study population. Therefore, we cannot exclude blood glucose as a significant mediator between adiponectin and MetS. Finally, the adipokines measured are only a small fraction of the wide array of pro- and anti-inflammatory biochemical indices that are produced by the adipose tissue.

This study has also strengths. Firstly, the measurement of biochemical indices was performed by a single certified laboratory. Secondly, the prevalence of MetS was evaluated by two definitions which are closely related and differ only by the abdominal obesity criterion. This allows for safer comparison between two existing definitions of MetS, and tests for the role of abdominal obesity in MetS. Furthermore, the adipokines measured were selected because of their proved and consistent association with adiposity, metabolism and inflammation. In addition, adiponectin and IL-6 were chosen to be measured because of their opposite functions and effects in metabolism and inflammation. Finally, this is one of the few studies which provide for a plausible explanation for the association between adiponectin and MetS.

## Conclusions

Adiponectin was identified as a significant and consistent parameter associated with MetS, whereas IL-6 was suggested to be only a biomarker of the MetS state. Moreover, MetS components which are indices of obesity and lipid metabolism (i.e. waist circumference, BMI, HDL-cholesterol and triglycerides), as well as insulin levels mediate, but do not abolish the association between adiponectin and MetS.

## Abbreviations

MetS: Metabolic syndrome
CVD: Cardiovascular disease
IL-6: Interleukin 6
IDF: International Diabetes Federation
AHA/NHLBI: American Heart Association/National Heart, Lung, and Blood Institute
BMI: Body mass index
HDL: High-density lipoprotein
SBP: Systolic blood pressure
DBP: Diastolic blood pressure
IQR: Interquartile range
OR: Odds ratio
95 % CI: 95 % confidence interval
ROC: Receiver operating characteristic analysis
AUC: Area under the ROC curve
